# Supplementary material for: Macrophages-induced IL-18–mediated eosinophilia promotes characteristics of pancreatic malignancy
Source: Life Sci Alliance. 2021 Jun 28;4(8):e202000979. doi: 10.26508/lsa.202000979 (PMC8321680; doi:10.26508/lsa.202000979)
Supplement: Supplementary file 2 [file LSA-2020-00979_TableS2.docx]

| **S.No.** | **Antibody** | **Catalog number** | **Supplier** | **Dilution** |
| --- | --- | --- | --- | --- |
| 1 | PDX-1 | sc-390792 | Santa Cruz Biotechnology, Inc. | 1:100 |
| 2 | SOX-9 | sc-166505 | Santa Cruz Biotechnology, Inc. | 1:1000 |
| 3 | MUC-2 | sc-7314 | Santa Cruz Biotechnology, Inc. | 1:200 |
| 4 | EPX | Clone (MM25.82.2.1) | Mayo Clinic | 1:5000 |
| 5 | VIP | sc-253347 | Santa Cruz Biotechnology, Inc. | 1:500 |
| 6 | TGF-β | sc-130348 | Santa Cruz Biotechnology, Inc. | 1:250 |
| 7 | SMAD4 | 46535 | Cell Signaling Technology | 1:500 |
| 8 | MBP | Clone (MT-14.7) | Mayo Clinic | 1:7000 |
| 9 | α-SMA | 19245 | Cell Signaling Technology | 1:600 |
| 10 | SPRR1A | ARP63528_P050 | Aviva Systems Biology | 1:500 |
| 11 | KRAS | 53270 | Cell Signaling Technology | 1:500 |
| 12 | p53 | sc-126 | Santa Cruz Biotechnology, Inc. | 1:500 |
| 13 | VEGF | sc-7269 | Santa Cruz Biotechnology, Inc. | 1:500 |
| 14 | TTF | sc-53136 | Santa Cruz Biotechnology, Inc. | 1:500 |
| 15 | Goat Anti-Mouse IgG | BA-9200 | Vector Laboratories, Inc. | 1:250 |
| 16 | Goat Anti-Rat IgG | BA-9400 | Vector Laboratories, Inc. | 1:250 |
| 17 | Goat Anti-Rabbit IgG | BA-1000 | Vector Laboratories, Inc. | 1:250 |

**Supplementary Table.2.**
