# Supplementary material for: Macrophages-induced IL-18–mediated eosinophilia promotes characteristics of pancreatic malignancy
Source: Life Sci Alliance. 2021 Jun 28;4(8):e202000979. doi: 10.26508/lsa.202000979 (PMC8321680; doi:10.26508/lsa.202000979)
Supplement: Supplementary file 3 [file LSA-2020-00979_TableS3.docx]

| **S.No.** | **Antibody** | **Catalog number** | **Supplier** | **Dilution** |
| --- | --- | --- | --- | --- |
| 1 | F4/80 | 30325 | Cell Signaling Technology | 1:50 |
| 2 | NLRP3 | 15101 | Cell Signaling Technology | 1:100 |
| 3 | KRAS | 53270 | Cell Signaling Technology | 1:100 |
| 4 | p53 | sc-126 | Santa Cruz Biotechnology, Inc. | 1:100 |
| 5 | CD163 | 333605 | BioLegend | 1:100 |
| 6 | CD11b | 11-0112-41 | Thermo Fisher Scientific | 1:100 |
| 7 | CD86 | 105007 | BioLegend | 1:100 |
| 8 | Streptavidin, Alexa Fluor™ 594 | S32356 | Thermo Fisher Scientific | 1:400 |
| 8 | Goat anti-Rabbit IgG Alexa Fluor Plus 488 | A32731 | Thermo Fisher Scientific | 1:400 |
| 10 | Goat anti-Mouse IgG Alexa Fluor Plus 488 | A32723 | Thermo Fisher Scientific | 1:400 |

**Supplementary Table.3.**
