# Supplementary material for: Macrophages-induced IL-18–mediated eosinophilia promotes characteristics of pancreatic malignancy
Source: Life Sci Alliance. 2021 Jun 28;4(8):e202000979. doi: 10.26508/lsa.202000979 (PMC8321680; doi:10.26508/lsa.202000979)
Supplement: Supplementary file 4 [file LSA-2020-00979_TableS4.docx]

| **S.No.** | **Antibody** | **Catalog number** | **Supplier** | **Dilution** |
| --- | --- | --- | --- | --- |
| 1 | NLRP3 | 15101 | Cell Signaling Technology | 1:1000 |
| 2 | IL-18 | orb539965 | Biorbyt | 1:1000 |
| 3 | GAPDH | 2118 | Cell Signaling Technology | 1:1000 |
| 4 | TGF-β | sc-130348 | Santa Cruz Biotechnology, Inc. | 1:500 |
| 5 | SMAD4 | 46535 | Cell Signaling Technology | 1:500 |
| 6 | β-actin | 3700 | Cell Signaling Technology | 1:3000 |
| 7 | SPRR1A | ARP63528_P050 | Aviva Systems Biology | 1:1000 |
| 8 | KRAS | 53270 | Cell Signaling Technology | 1:500 |
| 9 | p53 | sc-126 | Santa Cruz Biotechnology, Inc. | 1:500 |
| 10 | CDK9 | 2316 | Cell Signaling Technology | 1:1000 |
| 11 | CDKN2A | 10883-1-AP | Proteintech Group Inc. | 1:1000 |
| 12 | p-ERK | 4370 | Cell Signaling Technology | 1:1000 |
| 13 | ERK | 4695 | Cell Signaling Technology | 1:1000 |
| 14 | p-AKT | 4060 | Cell Signaling Technology | 1:1000 |
| 15 | AKT | 9272 | Cell Signaling Technology | 1:1000 |
| 16 | p-EGFR | sc-377547 | Santa Cruz Biotechnology, Inc. | 1:500 |
| 17 | Fibronectin | ab2413 | Abcam | 1:2000 |
| 18 | VEGF | sc-7269 | Santa Cruz Biotechnology, Inc. | 1:1000 |
| 19 | EPX | Clone (MM25.82.2.1) | Mayo Clinic | 1:1000 |
| 20 | MRC1 | I8704-1-AP | Proteintech | 1:1000 |
| 21 | pNLRP3 | MBS9430199 | MyBiosource.com | 1:1000 |
| 22 | Caspase1 | 24232 | Cell Signaling Technology | 1:1000 |
| 23 | IL-1β | 12426 | Cell Signaling Technology | 1:1000 |
| 24 | Anti-rabbit IgG, HRP | 7074 | Cell Signaling Technology | 1:3000 |
| 25 | Anti-mouse IgG, HRP | 7076 | Cell Signaling Technology | 1:3000 |

**Supplementary Table.4.**
