# Supplementary material for: Macrophages-induced IL-18–mediated eosinophilia promotes characteristics of pancreatic malignancy
Source: Life Sci Alliance. 2021 Jun 28;4(8):e202000979. doi: 10.26508/lsa.202000979 (PMC8321680; doi:10.26508/lsa.202000979)
Supplement: Supplementary file 5 [file LSA-2020-00979_TableS5.docx]

| **S.No.** | **Age** | **Sex** | **Pathology** |
| --- | --- | --- | --- |
|  | 81 | M | Carcinoma of pancreas |
|  | 66 | M | Carcinoma of pancreas |
|  | 77 | F | Carcinoma of pancreas |
|  | 53 | F | Carcinoma of pancreas |
|  | 69 | M | Carcinoma of pancreas |
|  | 80 | F | Carcinoma of pancreas |
|  | 72 | M | Carcinoma of pancreas |
|  | 59 | M | Carcinoma of pancreas |
|  | 61 | F | Carcinoma of pancreas |
|  | 55 | M | Carcinoma of pancreas |
|  | 65 | M | Carcinoma of pancreas |
|  | 43 | F | Uterine fibroid polyp |
|  | 53 | F | Hashimoto thyroiditis |
|  | 57 | F | Lymphoepithelial cyst of mouth |
|  | 52 | F | Polycystic kidney disease, adult type |
|  | 27 | M | Simple renal cyst |
|  | 57 | M | Benign prostatic hyperplasia |

**Supplementary Table.5.**
